# Mast Cells in the Brain: Enduring Mysteries, Emerging Roles

**DOI:** 10.3390/cells15090767

**Published:** 2026-04-24

**Authors:** Shivani Mandal, Paul Forsythe

**Affiliations:** Division of Pulmonary Medicine, Department of Medicine, Faculty of Medicine & Dentistry, and Alberta Respiratory Centre, University of Alberta, Edmonton, AB T6G 2B7, Canada; mandal2@ualberta.ca

**Keywords:** mast cell, brain, neuroinflammation, blood–brain barrier, immune surveillance, glial interaction, neuronal interaction, sleep, behavior, migraine

## Abstract

Mast cells are heterogeneous, tissue-resident immune sentinels best known for their roles in allergy and peripheral inflammation. The discovery of mast cells within the meninges and brain parenchyma over a century ago raised enduring questions regarding their function in the central nervous system (CNS), their ontogeny, and distinction from peripheral counterparts. Brain mast cells are sparse and predominantly located in perivascular niches rather than forming dense aggregates, a feature that has made them difficult to study. Nevertheless, accumulating evidence implicates mast cells in diverse aspects of CNS physiology and pathology, including regulation of blood–brain barrier (BBB) permeability and neurovascular function, as well as immune surveillance in contexts of infection and injury. The ability of mast cells to communicate with neighboring glial and neuronal networks suggests potential roles in modulating neural activity, development, and behavior, although this dimension remains incompletely understood. Much of the foundational literature predates advanced immunological tools, contributing to persistent misconceptions regarding the identity and significance of brain mast cells. In this review, we outline the history of research investigating this enigmatic aspect of mast cell biology, clarifying what is known, what remains speculative, and how emerging insights may help redefine the boundaries between classical immunology and neuroscience.

## 1. Introduction

Mast cells are a heterogeneous population of long-lived tissue-resident mononuclear myeloid cells characterized by numerous small secretory granules. These cells are found in nearly all tissues, often in the vicinity of epithelia, blood and lymphatic vessels, and nerves. The primary functions of mast cells include innate defense against diverse threats and regulating multiple aspects of adaptive immunity, including antigen presentation and lymphocyte recruitment [1].

Cells with mast cell-like characteristics emerged >500 million years ago in Ascidians (sea squirts), where two histamine and heparin-containing granulated cell types—circulating haemocytes and Test cells (found in the perivitelline space of oocytes)—provide innate immune protection [2]. However, actual mast cells most likely appeared 450–500 million years ago in the last common ancestor we share with hagfish, lamprey, and shark [3]. Over time, mast cells evolved to acquire multiple functions, playing roles in wound healing, coagulation, and defense against toxins [4,5,6]. Indeed, the persistence of mast cells throughout vertebrate evolution is a testament to the beneficial role these cells played well before the emergence of adaptive immunity and IgE, both of which are key to the role most associated with mast cells: drivers of allergic inflammation [7].

Like other tissue-resident immune cell lineages (e.g., macrophages), mast cells first originate from fetal-restricted progenitors [8]. Mast cells have dual hematopoietic origins and are produced in two waves during development. The first mast cells originate from a single wave of erythro-myeloid progenitors (EMP) that are produced by the yolk sac in a continuous manner starting on embryonic day seven [9]. These cells are supplemented with a second wave through the fetal liver. This second wave of cells consists of adult hematopoietic stem cell (HSC)-derived mast cell progenitors, which are directly recruited into peripheral tissues. In addition, some of these HSC progenitors migrate to the bone marrow, where they contribute to the long-term maintenance of mast cell populations [10]. While mast cells in connective tissues largely self-maintain thereafter, mucosal mast cells receive further supply from the bone marrow population after birth. In the course of time, fetal-derived HSCs are replaced with bone marrow-derived progenitors [11].

Mast cell progenitors migrate to tissues where they mature under the influence of local factors. Various integrins, chemokines, and cytokines regulate mast cell migration and homing to specific tissues during both normal and pathological states. Examples of such local factors include CXCL12, IL-4, IL9, IL-10, IL-31, IL-33, NGF, TGF-β, and most critically, stem cell factor (SCF), which, acting through c-kit (CD117), is essential for mast cell development, survival, and localization [12].

The maturation of mast cells under the influence of tissue-specific environments gives rise to tissue-specific mast cell phenotypes with distinct responses to stimuli and a unique assemblage of preformed and de novo-synthesized mediators [13]. Conventionally, mast cells in rodents have been categorized into two broad phenotypes: connective tissue (CTMC) and mucosal mast cells (MMC). MMCs are found mostly in the mucosa of the gut and lungs, where they reside between epithelial cells to defend against pathogens at host-environment interfaces. MMCs are often characterized as containing the protease tryptase, but not chymase. CTMCs are found in areas below the epithelium in connective tissue surrounding blood cells, smooth muscle, mucous, and hair follicles and are tryptase and chymase-positive. In humans, mast cells have been classified primarily based on protease content: mast cells that contain tryptase (MCT) or chymase (MCC) or tryptase and chymase (MCTC) [14]. However, these classifications do not do justice to the heterogeneity of mast cell populations both between and within tissues [15].

Given the tissue-specific heterogeneity of mast cells and the highly regulated and “immune privileged” environment of the central nervous system (CNS), it is perhaps not surprising that evidence suggests mast cells in the brain exhibit unique phenotypes distinct from those found in other tissues. However, our understanding of these brain-specific mast cells and their roles within the CNS remains poorly defined, and findings across studies are fragmented and often contradictory. This review seeks to critically consolidate current knowledge (Table 1), highlight unresolved controversies, and propose specific avenues to clarify how mast cells shape brain physiology and pathology.

## 2. Discovery of Mast Cells in the Brain

The earliest description of mast cells dates back to 1863, when Von Recklinghausen observed granulated cells in the frog mesentery [16]. Waldeyer later referred to these as “plasma cells” [17], but it was Paul Ehrlich, as a medical student in Freiburg, who recognized their unique staining properties and named them Mastzellen—“well-fed cells”—based on the erroneous assumption that their dense granules reflected phagocytic activity. Using aniline dyes, Ehrlich highlighted their metachromasia and noted the cells were frequently localized around blood vessels, nerves, and ducts [18], foreshadowing the diverse roles in which they would later be implicated [19].

Through the late 19th and early 20th centuries, mast cells were increasingly documented across animal species and pathological conditions [20]. Within the CNS, they were first reported in the meninges [21], in ischemic infarcts, and in the human plaques of multiple sclerosis (MS) [22,23]. Skepticism initially surrounded these findings: the brain was considered immune-privileged, and inconsistent reports—some failing to detect mast cells at all—fuelled doubt about the existence of the cell in the CNS [24,25]. Interest was renewed after mast cells were identified as major sources of histamine [26] and, in rodents, serotonin (5-HT) [27], production of which had previously been attributed primarily to neuronal sources. Observations that mast cells were a source of these neuromodulators prompted systematic surveys across multiple species to determine whether the cells contributed to production within the CNS.

In the 1970s, Ibrahim and Dropp conducted comparative studies across 104 mammalian species that established mast cells did exist in the brain but are highly variable in number and distribution [24,28,29,30,31,32,33,34,35]. Variability was observed across species, between sexes [35,36] and even within and between litters of rats [37]. Persinger further emphasized that technical factors such as fixation or anesthesia did not explain this inconsistency, suggesting that the variability reflects genuine biological heterogeneity [38]. Reports also described age-related declines in mast cell numbers and sex-related differences, though these early observations were often difficult to interpret given limited sample sizes and methodological constraints [39].

Attempts to classify brain mast cells added further complexity. Ibrahim proposed two populations: “type 1” cells resembling classical mast cells [24] and “type 2” cells—later termed neurolipomastocytoids (NLMs)—characterized by polymorphic shape, higher lipid content, and paler granules [29,40]. Cammermeyer and others reported additional atypical forms with pale cytoplasms [30,41] or altered staining properties, while some mast cells in specific brain regions appeared histologically distinct, staining only with toluidine blue and lacking detectable histamine [42].

These early taxonomies suggested distinct subpopulations in the CNS, but whether such differences reflected true functional subsets, developmental intermediates, or staining artifacts remained unresolved. By the close of these early efforts, it was clear that mast cells do exist in the brain, but in numbers and phenotypes that are highly variable and context-dependent. However, there was little understanding of the mechanisms underlying this variability or, indeed, the role of the brain mast cell.

## 3. Origin and Distribution of Brain Mast Cells

Studies of brain development in rodents suggest that mast cells first appear in the pia mater, choroid fissure, choroid plexus, cortex and peri-hippocampal regions (edges of the choroid fissure, an extension of the ventricular system and directly adjacent to the developing hippocampus) [43] during late embryonic and early postnatal stages. These sites may serve as entry points for circulating mast cell progenitors from the periphery [44,45], as they lack a fully developed BBB [46]. Consistent with this hypothesis, peri-hippocampal mast cells that emerge during early development in mice exhibit evidence of local proliferation, a feature characteristic of progenitor cells. These peri-hippocampal mast cells largely disappear between postnatal days 10 and 20 [43]. Similar patterns have been reported in rats, in which mast cells present in the meninges early in development and decline after approximately postnatal day 20 [44,47,48].

However, brain mast cell populations may not exclusively arise from early immature progenitors, as studies in rats have shown that mature mast cells may also be capable of directly migrating into the healthy brain [49]. In adult rodent brains, mast cells remain perivascular, often clustering near large vessels but also appearing in association with smaller capillaries around astrocytic endfeet. They are observed near both venous and arterial vessels [42,50].

**Figure 1:**
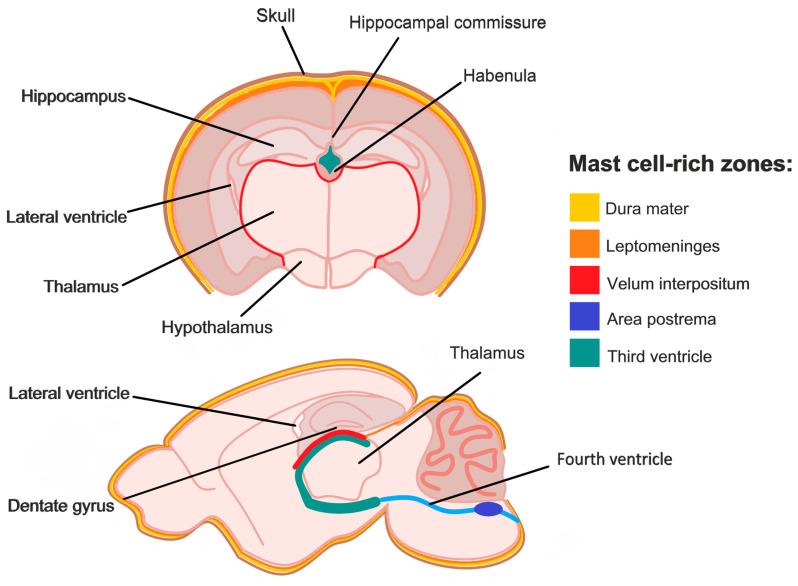
Brain mast cell distribution. Dural mast cells are primarily located in perivascular regions of the dura mater, the outermost meningeal layer enveloping the brain. Parenchymal mast cells are primarily distributed along blood vessels in regions that are in close proximity to the velum interpositum, including the habenular commissure, thalamus, hippocampal formation, and the choroid plexi of the lateral ventricles. They are also present in and around the third and fourth ventricles (area postrema). The velum interpositum and ventricular system are interconnected.

In healthy adult mice, the total number of brain mast cells is estimated to be approximately 300–500, with substantially higher numbers observed during development that gradually decline with age [51]. The majority of mast cells reported in mice are located in the meninges and the velum interpositum [25,37,43,52,53,54,55,56,57], although rare mast cells have been observed in other brain regions—including the cortex, olfactory bulbs, amygdala, and cerebellum [58]. It is important to note that many rodent studies describe mast cell accumulation in the sub-arachnoid space extending from the third ventricle toward the lateral ventricle and choroid plexus beneath the hippocampus going along the thalamus and hypothalamus, but refer to this region using different terms, such as peri-hippocampal space, below hippocampal fissure, hippocampal–thalamic border, or edge of the choroid fissure, habenular commissure, ventral thalamus and hypothalamus border. These descriptions largely refer to the same anatomical structure, the velum interpositum (VI) [59]. Mast cells are distributed along blood vessels in VI that perfuse or drain the thalamus, hippocampal formation, corpus striatum, corpora quadrigemini, and the choroid plexi of the lateral ventricles (Figure 1). Notably, the VI has been identified as a site of myeloid cell trafficking into the brain [60], offering the intriguing possibility that it may also serve as a route for mast cell entry into the CNS.

In rats, the Sprague Dawley strain exhibits both sex differences and hemispheric asymmetry, with females showing broader distribution—including the medial habenula and corpus callosum—while males display concentrations in the VI and meninges [61]. Other strains, such as Lewis and Wistar rats, present their own distinct patterns of mast cell distribution in the brain [62,63], suggesting that genetic background also influences the localization of these cells.

Mast cells have rarely been reported in the healthy spinal cord but have been found near the vessels and cells within the dorsal root ganglia and spinal roots in rodents [64].

In humans, mast cells have been identified predominantly within the meninges and in perivascular regions of circumventricular organs (CVOs) that lack a fully functional BBB, including the median eminence, pineal gland, area postrema, and subfornical organ. Mast cell density in these regions appears to peak during childhood and adolescence (up to approximately 19 years of age) [36] and then progressively declines with aging. In adults, mast cell density is typically low, with fewer than five mast cells per 5 µm thick tissue section in the meninges and perivascular regions. However, during viral, bacterial, or parasitic infections, mast cell numbers increase substantially, reaching approximately 11–20 cells per 5 µm section in the meninges and 5–20 cells in perivascular areas [65].

Reports of sex differences in mast cell distribution and responsiveness have been described in both rodents and humans, with males having higher numbers, especially during birth and development [36,66,67]. Importantly, brain mast cell numbers and localization are dynamic, shifting in response to circadian cycles [68,69,70], sleep cycles [71,72], hormonal fluctuations [67,73,74,75], mating behavior [55,56,67], environmental stimuli (magnetic fields [62,76], tactile stimulation [77,78]), vagal nerve activity [79,80], and injury.

Although there is considerable variability in mast cell numbers—likely reflecting their high sensitivity to local CNS environmental conditions, rapid responsiveness, and reported sex differences—a synthesis of available rodent data suggests that mast cell distribution is more consistent than often assumed—in the VI (Table 1). In humans, mast cell distribution appears to be more variable; however, the limited number of available studies precludes firm conclusions.

## 4. Brain Mast Cell Phenotypes

**Figure 2:**
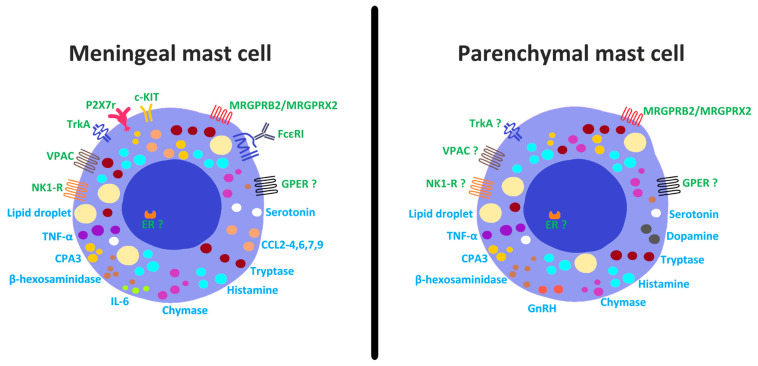
Known receptors (green) and mediators (blue) identified in brain mast cells. Expression varies across mast cell populations depending on location, stimuli, and functional state. A question mark indicates that there is some evidence for the presence of the receptor or mediator in brain mast cells, but definitive confirmation is lacking. Abbreviations: MC, mast cell; c-Kit, KIT proto-oncogene receptor tyrosine kinase (CD117); MRGPRB2/MRGPRX2, Mas-related G-protein-coupled receptors B2 (mouse)/X2 (human); FcεRI, high-affinity immunoglobulin E receptor; GPER, G protein-coupled estrogen receptor; CCL2/4/6/7/9, C-C motif chemokine ligands 2, 4, 6, 7, and 9; IL-6, interleukin-6; TNF-α, tumor necrosis factor alpha; CPA3, carboxypeptidase A3; NK1R, neurokinin-1 receptor; VPAC, vasoactive intestinal peptide receptor; TrkA, tropomyosin receptor kinase A (nerve growth factor receptor); P2X7r, purinergic receptor P2X7; ER, estrogen receptor; GnRH, gonadotropin-releasing hormone; and IBA1, ionized calcium-binding adaptor molecule 1.

Mast cell phenotypic heterogeneity has been attributed to multiple factors, including progenitor origin, developmental stage and interactions with the local microenvironment [81]. Interactions with organ-specific stromal cells have been proposed to play a dominant role in shaping mast cell phenotype through secreted factors such as SCF and IL-33 [82]. While there is evidence supporting this in the meninges [83], its relevance within the brain parenchyma is less straightforward. Mixed populations of c-Kit–positive and c-Kit–negative mast cells have been reported in the dura and leptomeninges [84,85,86,87]; however, parenchymal mast cells in adult rodent studies largely lack c-Kit expression [52,85,88] (Figure 2) despite c-Kit being the canonical receptor for SCF. Some research has suggested that SCF/c-Kit signaling may be dispensable for mast cell survival [89], a possibility that may be particularly relevant for brain mast cells. Alternative regulatory pathways involving nerve growth factor (NGF) and its receptor, tropomyosin receptor kinase A(TrkA) [90], sex hormones—particularly estrogen [90,91]—and signals derived from astrocytes [92], which have a close spatial association with parenchymal mast cells, may play a role in their maturation.

A similar pattern is observed with FcεRIα, which encodes the α-chain of the high-affinity IgE receptor. Peripheral mast cells consistently express this receptor, reflecting their classical role in allergen detection. In contrast, brain mast cells again exhibit regional heterogeneity, with mixed FcεRIα-positive and -negative populations in the meninges but predominantly absent expression within the parenchyma [88] (Figure 2) and choroid plexus [93] except under pathological conditions [94]. Notably, expression of both c-Kit and FcεRIα is more frequently observed in mast cells present in the parenchyma during early developmental stages, with these markers diminishing or disappearing with age [43].

Among regional brain populations, dural mast cells partially resemble peripheral CTMCs (tryptase+ chymase+) [91], though their developmental trajectory passes through mucosal-like intermediates, such that mucosal-type, mixed, and connective tissue-type phenotypes can coexist within the dura [48,95]. These cells contain histamine, heparin, tryptases [50,96,97,98], and chymases—Cma1 (human), mMCP-4 (mouse) [96,99], and rMCP-1 and rMCP-2 (rat) [53,95]. Transcriptomic analyses distinguish dural mast cells from peripheral CTMCs by their enrichment in cytokines such as TNFα, IL-6, chemokines including CCL2, CCL3, CCL4, CCL6, CCL7, and CCL9 [100], and high expression of histidine decarboxylase (Hdc) [96]. Mast cells in the dura mater highly express Mas-related G-protein-coupled receptors; Mrgpr2 (mouse) [101], MrgprB3 (rat) [43,102], and MRGPRX2 (human) [84] that mediate non-IgE-dependent degranulation by basic secretagogues and neuropeptides. However, MRGPR expression has not been confirmed on mast cells in the brain parenchyma.

Mast cells within the parenchyma differ markedly from peripheral tissue mast cells. During early postnatal life, a transient peri-hippocampal population (phMCs) emerges, proliferating locally as shown by BrdU (Bromodeoxyuridine) labeling. These cells express Cma1, c-Kit, and FcεRIα with varying ratios, consistent with heterogeneity in maturation states, and display a transcriptomic signature enriched for genes linked to neurodevelopment, including IGFBP1 and IGFBP2 (growth regulation), CYP26A1 and CYP26C1 (retinoic acid metabolism), PTGDS (eicosanoid synthesis), and CSF2 (microglial development) [43].

The mediator content of mast cells within the adult brain parenchyma has received very limited study. Nevertheless, existing evidence suggests that, in addition to histamine and tryptase, brain mast cells contain chymase, carboxypeptidase-3, TNFα [103,104], serotonin, dopamine, lipids, β-hexosaminidase [105] and heparin [106,107,108] (Figure 2). In adulthood, parenchymal mast cells appear to adopt phenotypes closely aligned with their local microenvironment. For example, a subpopulation of murine mast cells in proximity to microglia have been reported to express IBA-1 [109], a marker predominantly associated with microglia, while hypothalamic and thalamic mast cells express gonadotropin-releasing hormone (GnRH) [110,111], a hormone largely restricted to hypothalamic, thalamic and pituitary neurons. Mast cells within the thalamus and corpus striatum likewise exhibit region-specific characteristics, including differences in histamine content, dopamine granules [106], lipid composition, responsiveness to NGF and vasoactive intestinal peptide (VIP) [23,112,113] (Figure 2).

In addition to regional specialization, mast cells display morphological variation associated with maturation and activation state. Immature brain mast cells differ from mature ones in their glycosaminoglycan composition: they contain poorly sulfated heparin precursors and chondroitin sulfate E, whereas mature cells are enriched in highly sulfated heparin [33,114]. Heparin and chondroitin sulfate E help form the anionic gel matrix of granules, allowing mast cells to store large amounts of bioactive mediators [115]. This biochemical distinction not only affects staining properties—immature cells are Alcian blue-positive, while mature cells are avidin and safranin-positive and metachromatic with toluidine blue [20,48,90,116] but also underlies their capacity to store large amounts of bioactive mediators within granules. Morphologically, cells may appear densely granulated with well-defined borders under resting conditions, partially degranulated with diffuse contours during activation, or adopt pseudopodia with cytoplasmic extensions in response to local cues within the CNS [99]. Electron microscopy has revealed two main morphologies: elongated fusiform cells with irregular granules, and spherical cells with round nuclei and both dense and empty granules [117].

As these observations illustrate, mast cell identity is strongly context-dependent. Phenotypic differences are evident not only across tissues but also across species. For example, murine mast cells—both peripheral and central—synthesize and release serotonin [58,107,118], whereas human mast cells produce serotonin only under specific conditions, such as activation by substance P, and typically in much lower amounts [119,120]. Such interspecies and context-dependent differences complicate the reliable identification and characterization of mast cells and likely contribute to the historically inconsistent reports of their presence and phenotype in the brain.

It should also be noted that mast cells are distinct from other brain myeloid populations, including brain-associated macrophages (BAMs), microglia, dendritic cells and infiltrating monocytes, despite their shared myeloid ancestry [121]. In the brain, the most immediate distinctions are anatomical localization and metachromatic, granule-rich morphology. Marker-based discrimination is more challenging, however, because commonly used immune markers, including CD45, CD11b [122], CD68, c-Kit, FcεRIα and Iba1 [109], may show partial overlap among these cell types. In this regard, the increasing use of mast cell proteases such as carboxypeptidase A3 (CPA3), chymase and tryptase, along with other common markers, provides a more reliable means of distinguishing mast cells from other cells, as these markers are comparatively specific and remain robustly expressed across mast cell populations regardless of species, phenotypic state or brain location. While broad markers are useful for identifying the presence of mast cells, a better characterization of phenotypic diversity will likely provide insights into the functional role of mast cells in the brain.

## 5. Functions of Brain Mast Cells

Understanding mast cell function in the CNS is particularly challenging because much of the existing literature often extrapolates from models not directly related to the CNS—such as immortalized mast cell lines (HMC-1, LAD2, derived from mast cell leukemia) or from peripheral mast cell populations. While these approaches have been useful for generating hypotheses, as outlined above, brain-resident mast cells display distinct phenotypes and transcriptional profiles relative to peripheral counterparts [43], so inferences from non-CNSs must be interpreted cautiously and re-evaluated against brain-specific evidence.

Experimental constraints further complicate interpretation: a large portion of functional data comes from Kit-mutant “mast cell-deficient” mice, even though Kit is expressed across multiple cell types [123] and is essential for development and maintenance of neuronal populations [124,125]. KitW-sh/W-sh animals have pleiotropic abnormalities beyond mast-cell loss [126], so conclusions drawn from these models require confirmation in systems with intact Kit signaling. More selective genetic approaches have increasingly been adopted to address this limitation. These include the Cpa3Cre/+ model, in which mast cells (major Cpa3 producers) are ablated [127], and Mas-TRECK, which conditionally and selectively depletes Mcpt5/Cma1-expressing mast cells [128]. Yet even with these refined models, mast cells are eliminated throughout the body, and the current inability to selectively deplete or reconstitute the cells within the CNS limits mechanistic insight.

**Figure 3:**
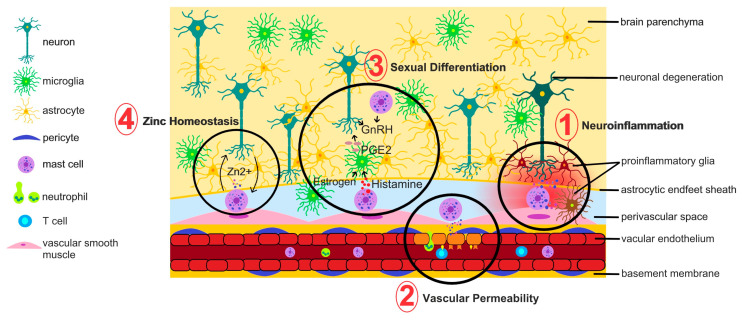
Proposed mechanistic functions of parenchymal brain mast cells for which at least a putative mechanism has been proposed. 1. Immune sentinels: Mast cells detect diverse injurious, immunological, or psychogenic stress signals. Upon activation, they release an array of preformed and newly synthesized mediators that influence nearby neurons and glial cells. These interactions can alter neuronal excitability, synaptic transmission, and glial cell states. Under pathological contexts, they can amplify glial reactivity and neuroinflammation. Such mechanisms are implicated in neuroimmune disorders, migraine pathophysiology and stress-induced behavioral alterations. 2. Regulation of vascular permeability: Mast cell-derived mediators can influence blood–brain barrier (BBB) integrity by increasing the expression of adhesion molecules on endothelial cells and destabilizing tight junction proteins. This results in increased vascular permeability and facilitates the recruitment of immune cells into the central nervous system. 3. Contribution to sexual differentiation: In the presence of elevated estrogen (E2), mast cells release increased levels of histamine. Together, estrogen and histamine stimulate microglia to release prostaglandin E_2_ (PGE_2_), which promotes gonadotropin-releasing hormone (GnRH) signaling in nearby neurons—an important process in sexual differentiation, including masculinization. GnRH-positive mast cells located near these neurons further contribute to the local pool of GnRH. 4. Regulation of zinc homeostasis: Mast cells may potentially influence labile zinc and zinc transporter levels in synaptic vesicles, thereby modulating neuronal synaptic transmission and plasticity.

Pharmacological tools also carry their own caveats: the “mast cell degranulator” C48/80 increases Ca^2+^ and spiking in multiple neuronal types [129] and can evoke histamine release from hypothalamic histaminergic neurons even without mast cells [130], whereas the “mast cell stabilizer” cromolyn modulates ion transport through its actions on chlorine and calcium channels [131,132], suppresses cytokine secretion in microglia [133] and reduces proinflammatory cytokine levels in mast cell-deficient mice [134], indicating mast cell-independent actions. Because the mechanisms of these agents are incompletely defined and they have broad impact, results obtained with them warrant careful scrutiny and, ideally, triangulation with newer genetic models. Here, we provide an overview of the functions (Figure 3) that have been specifically studied in the context of brain mast cells.

### 5.1. Contribution to Neuroinflammation

Inflammatory responses of the brain and spinal cord arising from disease, injury, infection or stress are collectively referred to as neuroinflammation. For many years, the brain was considered immune privileged, and neuroinflammatory processes were attributed primarily to resident glial cells, particularly microglia and astrocytes [135]. Glial cells were thought to mediate key inflammatory processes, including detection of injurious stimuli, rapid release of mediators to alert and recruit additional cells, modulation of vascular tone and permeability to permit cellular trafficking, and initiation of tissue repair [135]. However, accumulating evidence indicates that immune cells—both peripheral and resident—can also influence neuroinflammatory processes. In the following sections, we focus specifically on the growing evidence that several of these functions can be attributed to mast cells in the context of neuroinflammation.

#### 5.1.1. Immune Surveillance of the Brain

Resident microglia are widely regarded as the primary sensors and responders to infection and damage-associated signals within the CNS. But in addition to microglia, perivascular macrophages, dendritic cells, and T lymphocytes contribute to immune surveillance at the BBB, albeit at lower abundance [136]. Increasing evidence now suggests that mast cells, which are also found at the BBB, may contribute to early immune responses to these signals

Support for this view comes from experimental models of mild brain injury in mice, in which mast cells appear within hours of injury in the cerebral cortex, striatum, thalamus, and meninges, where they undergo degranulation and release histamine in both neonatal and adult animals [137,138]. Similar patterns of early mast cell accumulation in the parenchyma and degranulation within minutes to hours of induction have been reported in models of stroke [139,140], SARS-CoV-2 infection [141], Japanese encephalitis virus [105] and MS [142,143]—conditions characterized by tissue injury, infection, or aberrant immune activation. In several of these models, mast cell activation precedes astrocytic and microglial responses [144,145] as well as T cell and neutrophil infiltration into the brain. In addition, in a mast cell-deficient mouse model of MS, neutrophil influx was reduced and was restored following the reintroduction of mast cells into the brain, suggesting a role for mast cells in early immune cell recruitment [143,146].

Strikingly, in a mouse model of Alzheimer’s disease (AD), mast cells were reported to accumulate and respond to endogenous levels of soluble amyloid-β in the hippocampus and cortex, even before plaque formation and glial activation. These mast cells showed elevated basal hemichannel activity and persistent histamine release, mediated by amyloid-β-induced opening of Panx1 and Cx43 hemichannels in mast cells, with consequent calcium influx and degranulation [147]. This sustained release of histamine has been proposed as one factor that may contribute to the neuroinflammatory milieu observed in AD.

Evidence of immune surveillance from mast cells has not only been observed in brain parenchyma but also in the meninges. Interestingly, early mast cell responses to infection appear to contribute to host defense in the meninges, as studies report that mice lacking dural mast cells mount weaker immune responses to infection, accumulate higher bacterial [96] and viral loads in the brain [83] and show reduced recruitment of immune cells.

Collectively, these findings support a role for mast cells in early responses to pathogens and injury. This is in line with the established role of peripheral mast cells in sensing ‘danger cues’, instigating inflammatory responses and recruiting leukocytes to sites of tissue damage via the release of histamine. When activation is sustained, mast cells may further contribute to neuroinflammatory processes. However, the signals that drive their rapid recruitment and activation in response to stimuli remain unclear. The rapid responsiveness of mast cells may relate in part to their strategic localization within the meninges, where they are exposed to cerebrospinal fluid (CSF), and within the parenchyma, particularly in proximity to venous vessels where blood exits the brain. In these locations, mast cells are well positioned to sense signals originating from neural compartments, including danger-associated cues such as IL-33 released by damaged meningeal fibroblasts [83] or BBB cells, as well as allergens [148,149], and inflammatory mediators present in the CSF and circulation.

#### 5.1.2. Release of Mediators

Mast cells shape their local microenvironment through the release of a diverse repertoire of mediators. As immune sentinels, mast cells use these mediators to recruit and/or activate other immune and glial cells and to modulate vascular permeability, thereby facilitating further immune cell infiltration. In peripheral tissues, mast cells are known to release a wide range of mediators, including biogenic amines (histamine and serotonin); cytokines such as interleukins (IL-1–IL-6), leukemia inhibitory factor, tumor necrosis factor-α (TNF-α), interferon-γ, transforming growth factor-β, and granulocyte–macrophage colony-stimulating factor; enzymes including acid hydrolases, chymase, phospholipases, RMCP I and II, and tryptase; lipid mediators such as prostaglandins, leukotrienes, and platelet-activating factor; ATP; neuropeptides (e.g., VIP); growth factors such as NGF; nitric oxide; and proteoglycans including heparin [150]. Most of these have known functions in inflammatory processes [115]. However, it remains unclear whether brain mast cells release this full repertoire of mediators or whether the functional role of mediators is conserved within the CNS. Key unresolved questions include whether mediators such as eicosanoids, leukotrienes, ATP, VEGF, and NGF are released in the brain, and whether their release differs depending on the nature of the stimulus.

To date, only a limited subset of mast cell mediators—most consistently histamine, tryptase, and chymase—has been reliably detected in brain mast cells, highlighting a significant gap in our understanding of mast cell mediators and function in the CNS. Nevertheless, there is also evidence for the presence of additional mediators, including renin, vasopressin [151], TNF-α, prostaglandins, and IL-6, suggesting that the functional repertoire of brain mast cells may be broader than is currently appreciated.

These inflammatory mediators have been found to be stored within electron-dense cytoplasmic granules stabilized by an anionic gel matrix composed primarily of chondroitin sulfates and heparin [152].

It is also important to consider how mast cells release their mediators, as the mode of release can determine whether their effects are widespread and sustained or rapid and locally restricted [153]. Mast cells can release their mediators through multiple mechanisms, including full exocytosis, “kiss-and-run” exocytosis, piecemeal degranulation, and compound exocytosis [154]. The choice of release mechanism is highly dependent on the nature of the activating stimulus. For example, engagement of FcεRI typically induces the release of large granules in substantial amounts through compound exocytosis, resulting in relatively slow but robust signaling. In contrast, activation through MRGPR receptors triggers the rapid release of smaller granule contents [155], enabling more localized and transient interactions with the surrounding microenvironment. While compound exocytosis is the predominant mode of mast cell degranulation observed in peripheral tissues during allergic and inflammatory responses, piecemeal degranulation—more consistent with fine-tuned, localized signaling via small secretory vesicles [156]—appears to be the only consistently reported mode of mast cell mediator release within the brain [157].

#### 5.1.3. Regulation of Vascular Permeability

With the exception of CVOs, the brain vasculature is surrounded by pericytes, astrocytic endfeet, microglia and other cellular components that together form the BBB [158]. As noted above, mast cells are also located within this perivascular space. Their proximity to the vasculature places them in a position to influence BBB integrity and vascular permeability. Consistent with this, multiple in vivo studies have reported localized increases in BBB permeability in mast cell-rich regions, particularly following mast cell activation and degranulation [158,159,160,161].

The mechanisms by which mast cells influence the BBB are not yet fully defined. However, in vitro studies suggest that histamine, one of the major mediators released by mast cells, may contribute to changes in vascular function. Acting via H1 and H2 histamine receptors on endothelial cells [162], histamine activates phospholipase C and nitric oxide signaling pathways that promote endothelial cell contraction and transient opening of intercellular junctions, thereby increasing vascular permeability [163,164].

In addition, mast cell-derived mediators such as prostaglandins and tumor necrosis factor (TNF) [140,143] have been shown to alter endothelial transcriptional programs [165], resulting in upregulation of adhesion molecules including vascular cell adhesion molecule-1 (VCAM-1) and intercellular adhesion molecule-1 (ICAM-1). These adhesion molecules are critical for leukocyte adhesion to the endothelium and subsequent transmigration into the CNS, especially neutrophils [166]. In the brain, elevated VCAM-1 expression correlates with the severity of BBB disruption and vascular leakage [167], while increased ICAM-1 expression facilitates rapid CD4^+^ T cell trafficking across the BBB [168].

Other key mast cell-derived mediators that may contribute to BBB modulation are proteases like chymase [105] and tryptase. Tryptase has been shown to increase VCAM-1 expression in brain endothelial cells [169]. Tryptase can destabilize endothelial tight junction proteins, including claudins and occludin [170] through activation of endothelial protease-activated receptor-2 (PAR2), a mechanism that is well characterized in peripheral mast cell-rich tissues [171]. It has also been suggested that mast cells can release or indirectly increase gelatinases such as MMP-2 and MMP-9 [161,172]. MMP-2 and MMP-9 activation play an important role in BBB disruption.

Increases in BBB permeability induced by mast cell activation may reflect their role as immune sentinels in the brain, permitting immune cell infiltration, including by T cells and neutrophils, during infection or injury. Whether these effects are primarily protective or disruptive likely depends on the nature and duration of the inflammatory response.

### 5.2. Interaction with Glia

Microglia are resident macrophage-like cells of the CNS and play critical roles in CNS immunity and development. Upon activation, microglia exhibit a broad spectrum of responses ranging from pro-inflammatory to pro-regenerative states, positioning them as key regulators of neuroinflammatory processes [173].

Interestingly, mast cells are frequently positioned in close proximity to microglia [174] and studies suggest potential interactions between these two cell types in vivo [175]. As discussed above, mast cells function as surveillance cells and often respond rapidly to injury or infection in both the meninges and parenchyma by releasing mediators into their local environment. Several of these mast cell-derived mediators have been shown to interact with microglia. For example, mast cell-derived tryptase can activate PAR2 on microglia, triggering the release of IL-6 and TNF, reactive oxygen species and brain-derived neurotrophic factor (BDNF) via MAPK and NF-κB signaling pathways. In vivo, inhibition of this mast cell tryptase release has been shown to attenuate microglial activation, neuroinflammation, and hippocampal neuronal degeneration [176,177,178].

Mast cell-derived histamine also appears to modulate microglial activity. Microglia express histamine receptors H1–H4. In co-culture systems, mast cell-derived histamine has been shown to induce microglial production of prostaglandin E_2_ (PGE_2_) [67]. In rodent models, this interaction was confirmed as exposure to histamine promoted activation and, along with PGE_2_, release of TNF-α and IL-6 from microglia [179,180,181].

Beyond microglia, mast cells have also been proposed to interact with astrocytes, an idea supported by their localization near the astrocytic endfeet surrounding blood vessels, across species [182,183]. Although relatively few in vivo studies have examined mast cell–astrocyte interactions in detail [92], in vitro evidence indicates that astrocytes can be activated by mast cells through CD40–CD40 ligand interactions [184] and that mast cell-derived histamine can act on astrocytic histamine receptors (H1R and H2R) [185,186]. Conversely, cytokines released by astrocytes have been shown to induce mast cell degranulation [183], suggesting bidirectional communication.

### 5.3. Mast Cell Interactions with Neurons

Communication between mast cells and peripheral nerves is well documented [187,188]. Given their close spatial proximity in the CNS, analogous interactions within the brain are hypothesized [189] and there is some experimental evidence that lends support to this idea. Mast cell degranulation induced by C48/80 was demonstrated to influence neuronal activity in the thalamus of both male and female rats, with notable sex-dependent effects. Strikingly, in males, mast cell activation was associated with inhibitory effects on thalamic neurons, whereas in females it was associated with neuronal excitation. These 48/80-induced changes in neuronal activity were not observed in brain regions lacking mast cells, suggesting that the effects were mediated through mast cells rather than direct actions of the compound on neurons [190]. However, the mediators and signaling pathways responsible for these effects remain unresolved.

At present, histamine and tryptase are the two mast cell mediators most often considered candidate signals in such mast cell–neuron communication. It is known that C48/80 induces histamine and protease [191] release from brain mast cells. Thus, it is possible that mast cell-derived histamine and tryptase contribute to the observed neuronal modulation. However, the mechanisms by which these mediators act on neurons and the resulting neuronal responses appear to be highly context-dependent, which could also explain, at least in part, the observed sex differences. In vitro studies have shown that histamine can enhance NMDA receptor-mediated responses in cortical [192] and hippocampal neurons [193], thereby increasing neuronal excitability and, under some conditions, potentiating synaptically mediated excitotoxicity and neuronal injury [194,195]. Conversely, histamine can also exert neuroprotective effects by upregulating astrocytic glutamate transporters and glutamine synthetase, thereby reducing extracellular glutamate and protecting neurons from glutamate-induced toxicity [196]. Tryptase has likewise been proposed to influence neuronal function, potentially through PAR2 activation on neurons [197]. Evidence from both central and peripheral systems suggests that tryptase can alter neuronal excitability [178]. We also know that mast cell-derived tryptase does interact with enteric neurons in the gut [198], raising the possibility that similar mechanisms operate in the brain.

However, the effects of compound 48/80 should not be interpreted as evidence for histamine or tryptase alone. Compound 48/80 also induces the release of multiple mast cell mediators [151], like proteoglycans [199], and findings from hippocampal neuron coculture studies indicate that neuronal responses are unlikely to be driven by a single mast cell product in isolation. Rather, mast cell secretory products acting together can induce neuronal calcium signals and synaptic vesicle release in an NMDA receptor-dependent manner [199]. Thus, the neuronal modulation observed after mast cell activation may reflect the combined actions of several mediators rather than histamine and tryptase alone.

Some mast cells have also been reported to rapidly release GnRH [200,201], which appears to be taken up by neighboring neurons that do not typically express GnRH, by a process known as transgranulation [157]. Mast cell-derived GnRH may function as a local neuromodulatory signal linked to motivational and reproductive states, although direct evidence for this remains limited.

A further, less conventional route of interaction may involve zinc [202], a trace element that plays a critical role in neurogenesis, synaptic transmission, and neuronal plasticity [203,204,205]. In the brain, a small, free or “labile” pool of Zn is sequestered in synaptic vesicles, where it acts as an activity-dependent signaling ion that regulates vesicle release probability, synaptic plasticity, and postsynaptic excitability [206]. Of note, labile Zn is highly concentrated in the mossy fiber region of the dentate gyrus, an area that lies in close anatomical proximity to the velum interpositum, where mast cells are most abundant. Mast cell granules contain high levels of labile Zn, which is released upon activation and degranulation [207]. Although Zn efflux occurs alongside granule exocytosis, the signaling cascades governing Zn secretion are at least partly distinct from those that drive degranulation [208]. Mast cells also possess well-developed mechanisms for Zn uptake, allowing them to tightly regulate their intracellular Zn pool [209]. Given their capacity to both release and sequester the ion, mast cells have been proposed to contribute to local zinc homeostasis and thereby influence neuronal function. In this context, mast cell-deficient mice have been reported to have increased levels of labile Zn in the hippocampus compared to wild-type mice but no differences in total Zn in the whole brain or other tissues [206]. Furthermore, elevated expression of ZnT3, the transporter responsible for loading Zn into synaptic vesicles, was detected in the neuropil of the mossy fiber layer of the dentate gyrus of mast cell-deficient mice, again without changes in total zinc levels in the dentate gyrus [210]. Together, these observations suggest that mast cells may regulate the bioavailability and secretory vesicular concentration of Zn in the hippocampus, and that in their absence, hippocampal zinc homeostasis shifts.

This interaction may have broader implications for disorders in which Zn uptake and mast cell accumulation are altered, such as autism spectrum disorders, AD and mood disorders [211,212,213]. In these conditions, changes in mast cell density or activity could disrupt Zn regulation within the dentate gyrus and thereby perturb hippocampal signaling. Zn also plays a crucial role during early brain development [203], and mast cells have been reported to be particularly abundant during this period (see Section 3). It is therefore conceivable that these observations are mechanistically linked. Further investigation is therefore needed to clarify the role of mast cells in maintaining Zn homeostasis and to determine how these processes are influenced by alterations in mast cell number and/or activity.

Communication between mast cells and neurons is also likely to be bidirectional, with neurons providing signals that regulate mast cell phenotype and output. Among these, the neuropeptide pituitary adenylate cyclase-activating polypeptide (PACAP) is of particular interest. PACAP-expressing neurons are found in the hippocampus, amygdala, habenula, and various hypothalamic nuclei, including the ventromedial and mammillary nuclei [214]. PACAP binds to MRGPRB2 (or MRGPRX2 in humans), which is expressed in connective tissue mast cells [215] and has also been detected in meningeal mast cells. This interaction is said to contribute to mast cell degranulation, inflammatory mediators release and migraine-like pain [84] (Section 5.3). By contrast, VIP, which is also released by neurons and for which some brain mast cells express receptors, may have different effects [113,216]. VIP has been shown to interact with mast cells and promote the production of NGF. Given that NGF is a key trophic factor supporting neuronal survival, differentiation, and growth, this pathway suggests that neuron-derived signals may, under some conditions, shift mast cells toward a protective or modulatory phenotype rather than a disruptive one [112].

However, it is important to interpret findings from in vitro studies with caution. Some interactions observed between mast cells and neurons in controlled experimental settings have not been consistently replicated in vivo [217]. Direct evidence for such interactions in the brain remains limited, and many of the functional outcomes attributed to mast cells could also arise indirectly through mast cell interactions with glial cells, increased neuroinflammation, or changes in vascular permeability, which then alter neuronal response.

#### 5.3.1. Central Modulation of Pain

Mast cells are known to play a key role in peripheral pain associated with inflammation. Upon activation by injury, allergens, or neuropeptides, mast cells release mediators including histamine, tryptase, prostaglandins, and cytokines that trigger nociceptor (noxious-stimuli-sensing nerve endings) sensitization. Histamine and prostaglandins lower activation thresholds of TRPV1 (Transient Receptor Potential Vanilloid 1) on nociceptors, while tryptase cleaves PAR2 to enhance substance P release. Substance P can, in turn, promote further mast cell degranulation, creating a bidirectional feedback loop that sustains neurogenic inflammation and hyperalgesia. Growing evidence supports a similar role for meningeal and thalamic mast cells in central nociceptive processing, particularly in migraine pathophysiology [213], although in the CNS, they appear to function more as amplifiers than as primary initiators of pain. Direct electrical stimulation of the trigeminal ganglion activates meningeal nociceptors, which can evoke degranulation of adjacent dural mast cells through neuropeptide release. In turn, mast cells release mediators such as histamine, serotonin (5-HT) and Prostaglandin I2 (PGI2) that lead to TRPV1-dependent sensitization of the same meningeal nociceptors and other trigeminovascular neurons. This increased excitability lowers their firing threshold and enhances ongoing activity, thereby contributing to prolonged migraine pain [218,219,220].

Parenchymal mast cells also appear to respond to pain. Rodent studies show increased mast cell infiltration in contralateral thalamic regions following painful stimuli (e.g., repeated abdominal wall puncture or unilateral spinal nerve ligation). Sex appears to influence this response, with females exhibiting more thalamic mast cell infiltration associated with greater sensitivity to neuropathic and inflammatory pain [51,221].

A MrgprB2/MRGPRX2-PACAP axis (Section 5.2) appears to be an important mechanism in the amplification of migraine pain. PACAP released from trigeminal nociceptors can act as an endogenous agonist of MrgprB2, thereby driving mast cell degranulation [222]. Dural PACAP has been demonstrated to induce MrgprB2-dependent mechanical facial allodynia and migraine-like responses [101]. Furthermore, repetitive stress exposure in mice increases plasma PACAP levels, which activate MrgprB2 on dural mast cells and lead to TRPV1-dependent sensitization of trigeminal ganglion (TG) neurons and headache-like behaviors; these effects are abolished in MrgprB2-deficient animals [101].

Substance P may also function as a neuropeptide amplifier of migraine pain, at least in part by promoting MrgprB2/MRGPRX2-mediated mast cell degranulation. Beyond MrgprB2/MRGPRX2, functional studies indicate that dural mast cells also express P2X7 purinergic receptors, which respond to ATP. ATP can enhance nociceptive signaling in trigeminal terminals both directly, through neuronal P2X receptors, and indirectly, through P2X7-dependent mast cell degranulation [223,224,225]. Dural mast cells can also be activated by Calcitonin Gene-Related Peptide (CGRP) [226], Corticotropin-Releasing Hormone (CRH) [227] and acetylcholine/carbachol [91], via their respective receptors, leading to degranulation and subsequent modulation of nearby nociceptor terminals.

Currently, monoclonal antibodies to CGRP are approved for migraine treatment, and prevention of mast cell degranulation is thought to contribute to therapeutic efficacy [228]. However, the evidence that mast cells are important amplifiers of migraine pain suggests that more direct targeting of neural-mediated mast cell activation, particularly through MrgprB2/MRGPRX2 and P2X7 receptors, may provide effective adjuncts to existing therapeutic strategies.

### 5.4. Mast Cells and Sleep

Accumulating evidence is suggestive of links between brain mast cells and sleep or circadian biology. Mast cell-deficient Kit-mutant mice exhibit alterations in sleep regulation and rapid eye movement (REM) architecture compared with wild-type controls, while intracerebral administration of compound 48/80 increases histamine levels in the lateral ventricles and promotes wakefulness in wild-type mice, but not in Kit-deficient or Mas-TRECK mice [71,229,230]. Histamine is a well-established wake-promoting neuromodulator that activates arousal circuits via H1 receptor signaling [230,231], suggesting that mast cells may contribute to sleep–wake regulation through histaminergic mechanisms.

Interestingly, continuous darkness was found to double mast cell numbers in the leptomeninges and dorsal lateral geniculate nucleus of rats, with levels returning toward baseline within five days of light reintroduction [72]. Given that rodents are nocturnal and undergo restorative processes during the light (rest) phase, prolonged darkness may disrupt sleep and induce stress, both of which are known to enhance mast cell activity [232]. As such, these observations may reflect stress-mediated effects on mast cells rather than direct circadian regulation. However, an alternative, albeit highly speculative, explanation involves the glymphatic system. Sleep is critical for efficient glymphatic function [233], a CNS-specific waste clearance pathway in which CSF circulates through perivascular spaces organized by astrocytic endfeet [234,235]. Mast cells sit at these endfeet in the perivascular spaces. It is therefore conceivable that mast cells may migrate within or monitor glymphatic flow [236]. During prolonged periods of sleep disruption, impairment of this flow could potentially contribute to mast cell accumulation within these regions. Mast cells may be capable of sensing changes in glymphatic dynamics, influencing perivascular CSF movement, or responding to the accumulation of metabolic waste products such as amyloid-β or lactate. Although speculative, this hypothesis raises the possibility that mast cells may play a previously unrecognized role in linking sleep, waste clearance, and immune surveillance in the CNS and warrants experimental testing.

### 5.5. Mast Cells and Behavior

The possibility that central mast cells may influence neuroendocrine and behavioral responses to stress and immune challenges was first highlighted by Rae Silver and colleagues in the 1990s. This stemmed from pioneering research demonstrating that GnRH-immunoreactive mast cell numbers surged in the medial habenula of ring doves following courtship, which identified mast cells as behavior-responsive “mobile sentinels” migrating into brain regions under gonadal steroid influence, and challenging the concept that brain immunity is static.

Further research extended these observations of behavior-responsive mast cells to mammals. Male mice that mate and subsequently cohabitate with females show elevated thalamic mast cell numbers compared with males housed with other males [55], with enrichment of GnRH-positive mast cells. Similarly, male rats cohabitating with ovariectomized but sexually receptive females exhibit increased thalamic mast cells relative to males housed with non-receptive ovariectomized females or other males [237]. In female prairie voles, exposure to male urine increases mast cell numbers in the main olfactory bulb and medial habenula, consistent with the reliance of the species on male-derived cues to trigger ovulation [75]. Mast cell numbers also fluctuate with ovarian development, pregnancy [49], masculinization, and androgen production [238,239]; contexts all characterized by dynamic changes in sex hormone levels.

In addition to responding to sex hormone-associated behaviors, there is also evidence that mast cells can influence behavior in selected developmental paradigms, although the strength of evidence varies across models. During the masculinization window of development, male rats exhibit elevated estradiol levels alongside increased mast cell numbers and histamine levels compared to females. Estrogen can stimulate mast cells to release histamine [67]. This increase in estradiol and mast cell-derived histamine can stimulate microglia to release PGE_2_, the induction of which is essential for masculinization [240]. Experimentally increasing estradiol in neonatal female rats elevates mast cell numbers in the preoptic area (POA), a key node for copulatory and maternal behaviors, with an associated masculinization of behavior [67]. Conversely, inhibiting mast cell activity during development reduces male-typical copulatory behavior in adulthood [67]. Interestingly, prenatal stress produces similar effects. Female offspring of stressed mothers exhibit increased mast cell and microglia activation, as well as masculinization of dendritic spine density in the POA. In adulthood, these female offspring display elevated male-typical mounting behavior. In contrast, male offspring of stressed mothers show evidence of de-masculinization, including reduced microglia activation, decreased neonatal dendritic spine density, diminished male-typical copulatory behavior, and reduced olfactory preference for female-typical cues [241]. These findings suggest that mast cells may participate in neuroimmune pathways linked to developmental sexual differentiation.

Mast cells also appear to respond to psychological stressors. Early life adversity, such as maternal separation or early weaning, increases mast cell numbers, chymase levels and TNF expression in the dura of female mice but not males [99]. The effects of early-life stress in females persist into adulthood, where mild stress exposure similarly elevates chymase levels with associated anhedonia, as determined by reduced sucrose preference. Administration of the mast cell stabilizer, ketotifen, prior to adult stress exposure prevents both chymase upregulation and sucrose preference reduction, supporting a direct mast cell–chymase–anhedonia link [99]. In adult male mice, repeated social defeat increases mast cell numbers in the ventral thalamus, habenula, and hypothalamus (strongly suggesting mast cells in the velum interpositum) [242]. Social isolation has also been associated with increased mast cell numbers [243,244]. Short periods of restraint stress exposure also induce degranulation of dural mast cells, elevate mast cell proteases in CSF, and increase dural vascular permeability [227], effects that are absent in c-Kit -/- mast cell-deficient mice. Consistent with the sensitivity of mast cells to aversive stimuli, fear conditioning using electric foot shock increases mast cell numbers in the medial and lateral habenula of rats. Notably, mast cell accumulation and anxiety-like behavior are further exacerbated when fear-conditioned rats are housed in social environments with non-fear-conditioned conspecifics [245], suggesting a potential link between social context, fear processing, and mast cell activity [246]. In contrast, positive physical stimuli such as gentle handling and tactile stimulation reduce mast cell numbers in the leptomeninges and diencephalon [77,78].

Overall, converging evidence indicates that psychological stressors induce region-specific changes in mast cell numbers, implicating these cells in the modulation of stress- and affect-related circuitry. By remodeling the local neuroimmune environment, mast cells may alter subsequent tissue responsiveness and influence behavioral outcomes. However, the functional significance of numerical shifts in brain mast cells is unresolved. Depending on context, mast cells could either promote maladaptive stress signaling or serve as a compensatory, protective brake on excitability and tissue injury.

While there is compelling evidence that brain mast cells respond to salient psychosocial stimuli, little is known about the mechanisms underlying behavior-associated changes in mast cell number and activity. However, one study demonstrated that indicators of restraint stress-induced mast cell activation, along with associated increased dural vascular permeability, were absent in mice lacking the Neurokinin 1 receptor (NK1R). NK1R is expressed on mast cells and is a receptor for substance P. However, mast cell activation and associated physiological responses remained intact in substance P-deficient animals [247], suggesting that NK1R activation occurs independently of substance P, likely through alternative ligands such as hemokinin-1 and Neurokinin-A. Notably, an NK1R-dependent, substance P-independent activation of peripheral (dermal) mast cells, in response to restraint stress, has also been described [248]. NK1R is also expressed on glial cells, meaning caution is required in attributing effects of receptor deficiency specifically to mast cells. However, in the case of restraint stress-induced increase in dural vascular permeability, mast cell deficiency phenocopies NK1R loss, strongly suggesting a stress-NK1R-mast cell activation pathway [247].

There is also evidence for brain mast cell involvement in the regulation of complex behaviors often associated with stress exposure. Studies utilizing mice with a c-kit mutation (KitW−sh/W−sh) identified that mast cell deficiency was associated with increased anxiety-like behavior in exploratory paradigms, such as the elevated plus maze and open field test, as well as impaired social behavior [249]. The increased anxiety-like behavior observed with mast cell deficiency was replicated in wild-type mice by central, but not peripheral, administration of the mast cell stabilizer cromolyn. Correspondingly, an independent study demonstrated that cerebral administration of the mast cell degranulating agent, compound 48/80, increased exploratory and social behavior in wild-type mice but not in mast cell-deficient animals [250]. While studies in KitW−sh/W−sh should be interpreted with care, as these mice have additional abnormalities (including an increase in myeloid-derived suppressor cells, which are known to interact with microglia), changes in behavior, specifically reduced social interaction preference, have been replicated in a more recently developed and mast cell-specific knock-out model (Mas-TRECK) [251].

It is noteworthy that the current literature presents a paradoxical relationship between mast cells and anxiety-like behavior. While psychological stressors often increase mast cell number or activation in association with anxiety-like or anhedonia-like phenotypes, mast cell deficiency has also been linked to increased anxiety-like behavior and impaired social interaction. In the absence of direct mechanistic evidence, it remains unclear whether mast cell changes are causally responsible for these behavioral outcomes. However, a plausible explanation is that mast cells contribute to baseline homeostatic signaling required for normal behavioral regulation, such that both excessive activation and loss of mast cell function may be disruptive. Their reported involvement in processes such as zinc homeostasis may support this model, although direct links to behavior remain to be established.

Brain mast cells also appear to be involved in behavioral conditioning. In Pavlovian conditioning paradigms, animals that preferentially attend to reward-predictive cues display greater mast cell degranulation in the thalamus than animals that focus on reward delivery itself. Blocking mast cell degranulation before training reduces cue-directed behavior, suggesting that mast cell activity contributes to the development of cue–reward associations [252]. Other conditioning studies further support the idea that mast cell activation can become associated with learned sensory cues, highlighting a bidirectional interaction between mast cell activity and neural circuits underlying motivation and learning [253].

Clinical observations in mastocytosis and allergic disease further raise the possibility that excessive mast cell activity may be associated with behavioral and cognitive symptoms [254,255]. When considered together with the preclinical evidence outlined above, these findings raise the possibility that brain mast cells may represent a relevant target in stress-related and affective disorders [256]. At present, however, such a view remains provisional, and further work is required to define the specific mast cell receptors and mediators involved in modulating behaviors, along with the development of novel CNS available mast cell stabilizers.

## 6. Knowledge Gaps and Future Directions

Mast cells are now recognized as rare but strategically placed residents of the meninges, perivascular spaces, and select parenchymal regions. However, despite a long history of study, many questions raised by some of the earliest investigators of brain mast cells remain unanswered. The timing and routes of mast cell progenitor entry into the CNS, cell turnover and trafficking dynamics across the lifespan are still poorly understood. Other key unknowns include whether there are stable, brain-specific mast cell phenotypes distinct from peripheral counterparts and to what extent these cells shape synaptic plasticity, neurogenesis, and stress- or sex-dependent behavioral outcomes under physiological conditions. Another open question is whether brain mast cells participate in angiogenesis. This possibility has been hypothesized on the basis of their perivascular localization, and the pro-angiogenic potential of mast cell mediators, but direct evidence in the CNS remains lacking.

Mast cells in peripheral tissues function as surveillance cells that monitor the environment and alert the adaptive immune system. The presence of mast cells in the brain, where microglia fulfill the immune surveillance role, raises the question of what additional functionality these granulocytes provide. Current evidence suggests that brain mast cells fine-tune physiological processes while retaining rapid response functions to injury or infection. Their sensitivity to behavioral and environmental stimuli fits this role. It has been proposed, albeit speculatively, that such responsiveness could confer adaptive advantages in contexts like stress, infection control, pain, sleep, reward and exploratory behaviors, and sexual behaviors.

Functionally, mast cells may bridge the temporal gap in responsiveness between neurons and microglia. Neurons transmit signals within milliseconds but lack immune effector capacity, whereas microglia generate slower, sustained immune responses. Mast cells, capable of reacting within seconds, can therefore provide rapid, modulatory signals that link neural and immune activity. Meningeal mast cells resemble peripheral connective-tissue-type mast cells and have been proposed to help limit pathogen entry at the brain borders. By contrast, parenchymal mast cells appear more tightly regulated, possibly with downregulated FcεRI in the relative absence of antigenic/bacterial stimuli behind the BBB, and may rely on piecemeal degranulation and phenotype shifts rather than explosive degranulation.

This plasticity likely contributes to the heterogeneity reported across studies. Rather than antigen-specific “memory” (as in T or B cells), mast cells may display functional memory, tuning gene expression with repeated stimuli and local cues. For example, subsets involved in developmental or reproductive circuits have been reported to express GnRH, whereas dura-associated mast cells engaged in barrier or injury responses may retain FcεRI expression. Such context dependence is plausibly shaped by neuroendocrine signals and lipid/epigenetic regulators, including sphingosine-1-phosphate (S1P) and microRNAs.

Most knowledge about brain mast cells derives from animal studies, and how these findings translate to humans remains uncertain. Human data are limited. Mast cells and mast cell-associated genes have been identified in lesions, white matter, and CSF of patients with MS [257]. In addition, drugs already used in MS treatment, or currently under clinical investigation, including dimethyl fumarate [258], natalizumab, and masitinib [259], have been proposed to exert at least some of their effects through mast cell-related mechanisms. In AD, autopsy studies have reported numerous tryptase-containing mast cells in proximity to amyloid plaques in several brain regions [260]. Clinical observations in mastocytosis also suggest a possible link between mast cells and neuropsychiatric dysfunction [261]. Patients with mastocytosis frequently experience depression and anxiety [262]. Yet, despite mastocytosis being a mast cell-driven disorder, the degree to which neuropsychiatric symptoms of the disease are mediated by CNS-resident mast cells, versus peripheral mast cell mediators acting on the brain, remains unknown [254]. A key challenge will be to move beyond correlative observation in humans to causal, circuit-level understanding of how CNS-resident mast cells and peripheral mast cell mediators jointly shape neuroinflammation, barrier integrity, and behavior. Single-cell and spatial-omics approaches in human postmortem tissue could help define mast cell niches along the BBB, meninges, and parenchyma and provide indications of functional dialog with neurons, microglia, and astrocytes. In addition, parallel studies in humans and animal models could test whether mast cell inhibition (via drugs such as masitinib, cromolyn analogs, or other kinase/mediator blockers) shifts disease trajectories in both rodent neuroinflammatory readouts and human neuroimaging, cognitive, or mood endpoints. Ultimately, such translational work on brain mast cells should determine whether it is more efficacious to target these cells as “early responders”, regulating BBB breach and neuroinflammation, or later, as amplifiers of chronic neurodegeneration and mood/cognitive dysfunction.

Our knowledge of brain mast cells to date has been limited by the investigative tools available, but methodological advances that allow integration of advanced phenotyping, spatial mapping, functional manipulation and 3D cell culture models [263] provide new opportunities to address current unknowns. Single-cell and spatial multi-omics (transcriptomics and proteomics) applied to enriched brain immune fractions can define mast cell heterogeneity, developmental trajectories, and circuit-specific niches. In vivo two-photon and light-sheet imaging through cranial windows, including dural and meningeal preparations, can visualize mast cell–neuron–vasculature interactions and degranulation dynamics, while genetically targeted gain- and loss-of-function tools (e.g., Cre-driver lines, inducible ablation, opto/chemogenetic control of mast cell activation) overcome the limitations of traditional “mast cell–deficient” strains. Additional models, such as BBB-on-chip or organoid–meninges co-culture systems, can be developed for a deeper mechanistic understanding of brain mast cells. Such approaches may finally provide answers to some longstanding questions and, importantly, allow us to determine the potential of brain mast cells as tractable, mechanistically grounded targets in neurological disease that may inspire new therapies for neuroinflammatory, neurodegenerative, and stress-related disorders.

## Figures and Tables

**Table 1:** **Overview Of Brain Mast Cells:** Mast cell location (Section 3), phenotype (Section 4), proposed functions, and disease relevance in distinct brain compartments (Section 5). Please refer to the sections for references.

**Location**		**Meninges**	**Leptomeninges**	**Parenchyma**	**Ventricles and surrounding circumventricular organs**
		Duramater	Pia mater, Arachnoid Mater	cortex (rare), velum interpositum (subarachnoid) and parenchyma surrounding velum interpositum— hippocampus, thalamus, habenula, corpus callosum	lateral ventricle (choroid plexus), third ventricle (subfornical organ, median eminence, pineal gland), fourth ventricle (area postrema) Note: Velum interpositum is interconnected with the ventricular system
**Phenotype**		Cpa3+; mixed populations of MTC and MT and MC		Cpa3+; largely MTC	
		c-Kit and FcεRIα heterogenous	c-Kit and FcεRIα heterogenous	**early postnatal mast cells**	**adult mast cells**
				c-Kit+, FcεRIα+ with variable ratios;	low/negative c-kit and FcεRIα
		Strong Mrgpr2/MrgprB3/MRGPRX2 expression in mouse/rat/human dura;		neurodevelopmental and eicosanoid-related signaling. These markers decline with age as transient cells disappear.	some adopt local markers such as IBA-1 near microglia or GnRH in thalamic/hypothalamic regions
**Functions**	**Demonstrated**	immune surveillance; host defense; vascular permeability; migraine-like pain pathophysiology		immune surveillance; neuroinflammation; vascular permeability; stress responsive; masculinization and sexual differentiation	
	**Putative**	glymphatic clearance; angiogenesis	responsive to pathophysiological state; wakefulness	neurodevelopment; glial interaction; neuronal interaction; behavior modulation; zine homeostasis; neuroprotection; glymphatic clearance	
**Diseases/Disorders associated**		Migraine; bacterial meningitis; stroke/ischemia; multiple sclerosis; traumatic brain injury		Neuropathic pain; mood disorders; multiple sclerosis; stroke/ischemia; traumatic brain injury; neurodegenerative diseases; autism spectrum disorder	tumor-associated hydrocephalus

## Data Availability

No new data were created.
